# An evaluation of the Waters Pico-Tag system for the amino-acid analysis of food materials

**DOI:** 10.1155/S1463924686000330

**Published:** 1986

**Authors:** J. A. White, R. J. Hart, J. C. Fry

**Affiliations:** Leatherhead Food Research Association Randalls Road Leatherhead Surrey KT22 7RY UK


Journal of Automatic Chemistry/*Journal of Clinical Laboratory Automation*, Vol. 8, No. 4 (October–December 1986), pp. 170–177

### An evaluation of the Waters Pico-Tag system for the amino-acid analysis of food materials

**J. A. White, R. J. Hart and J. C. Fry**
*Leatherhead Food Research Association, Randalls Road, Leatherhead, Surrey KT22 7RY, UK*

### Introduction

The Pico-Tag method, recently described by Heinrikson and Meredith [1] and developed commercially by Waters Associates, is an integrated technique for amino-acid analysis. Phenylisothiocyanate (PITC, or Edman's reagent) is used for pre-column derivatization, while reversed-phase gradient elution high-performance liquid chromatography (HPLC) separates the phenylthiocarbamyl (PTC) derivatives which are detected by their UV absorbance. Figure 1 illustrates the chemistry of the derivatization reaction. Cohen, Tarvin and Bidlingmeyer [2] from Waters Associates have reviewed the potential of the Pico-Tag method.

The technique was developed to improve the speed and sensitivity of amino-acid analysis. This need for faster analysis times and greater sensitivity arises from the recent popularity of HPLC for rapidly separating very small amounts of complex peptides and protein mixtures.

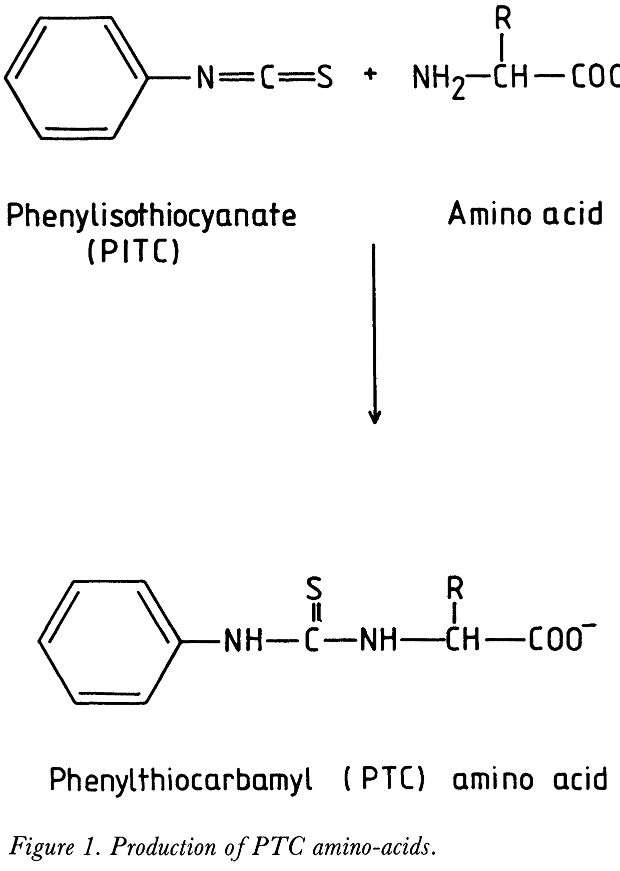

*Figure 1. Production of PTC amino-acids.*

The generation of numerous peptides and proteins for amino-acid analysis has thus placed a great strain on current instrumentation. Hence, there is a requirement for a fast and sensitive technique which does not waste valuable samples.

The traditional method of amino-acid analysis is separation by ion-exchange chromatography (IEC) developed by Moore, Spackman and Stein [3]. Samples are applied to the column at low pH, and eluted with a series of buffers of increasing pH and/or ionic strength. Post-column derivatization with ninhydrin then gives a product which is detected colorimetrically. The majority of current amino-acid analysers employ this technique.

The Pico-Tag method appears to have quite substantial advantages over the conventional ion-exchange analyser. The most important of these are speed, greater sensitivity, excellent reproducibility and general reliability of both the results and the hardware.

The separation of the PTC derivatives of the common amino-acids takes 12 min (total analysis time, including column regeneration time, is 20 min) with the Pico-Tag method, compared with about 90 min for a conventional ion-exchange analyser using sodium-based buffers.

The manufacturer claims very good reproducibility, which should allow automated data collection and calculation of results. With ion-exchange separation this is often not possible owing to variability of retention times.

One noteworthy feature of the analysis with PITC is the quantification of secondary amino-acids, such as proline and hydroxyproline. Unlike post-column ninhydrin detection or ortho-phthalaldehyde (OPA) derivatization, the proline and hydroxyproline PTC derivatives have the same chromophore and approximately the same molar response as other amino-acids. There is thus no need to suffer reduced sensitivity or to employ a second detector for these secondary amino-acids.

The Pico-Tag system can, with the appropriate column and solvent(s), also be used as a general-purpose HPLC, offering greater flexibility compared with the dedicated amino-acid analysers.

Conventional ion-exchange analysers using post-column derivatization are complex and hence sometimes temperamental instruments, usually with quite intricate fluidics and expensive electronics in close association. In contrast, the Pico-Tag system is of a simpler, modular design, allowing easy access to the individual units. Hence, the HPLC system should be more reliable mechanically.

The Pico-Tag system was originally developed and sold for the analysis of purified proteins. Detection of levels as low as 1 picomole is claimed by Waters, but this would rarely, if ever, be required for the application which we have investigated, namely food analysis.

### Description of the Pico-Tag system

The Pico-Tag system consists of the following:

(1) Two pumps for the delivery of eluents A and B.
(2) WISP autosampler for automated sample loading.
(3) System controller for automated control of system (programming, methods, pumps).
(4) Pico-Tag column (performing the separation).
(5) Column heater and temperature control module (maintaining the column temperature).
(6) UV detector, fixed wavelength at 254 nm (detection of PTC derivatives).
(7) Data module (combined plotter and integrator).
(8) Pico-Tag work station (for hydrolysis and pre-column derivatization of samples).
(9) Compressor (to operate the autoinjector system on the WISP).
(10) Two-stage rotary vacuum pump (user-supplied; required for Pico-Tag work station).

### Methods

Preparation of food samples for analysis on the Pico-Tag involves the following procedures, each of which is discussed in some detail in this section:

(1) Conventional hydrolysis, usually in acid, to release the constituent amino-acids (a general procedure is described).
(2) Preparation of the hydrolysate for chromatography, i.e. drying, re-drying and derivatization.
(3) Separation of the PTC derivatives by HPLC.

*Hydrolysis*

Microgram amounts of pure protein can be successfully hydrolysed in the small oven situated in the Pico-Tag work station. Foods, however, due to their mainly heterogeneous nature, cannot be sampled satisfactorily for hydrolysis in this way. A representative sample of a food may be 5 g or more (especially in the case of low-protein foods or liquid samples). Consequently, such materials are hydrolysed independently of the work station by the general method described here.

Food samples, equivalent to about 0·2 g of protein and norleucine (as internal standard), are weighed accurately in a 250-ml round-bottomed flask. The ratio required is approximately 15 mg protein to 1 mg norleucine, which may be added conveniently as a solution of known concentration, typically 1 g/1, in 6N hydrochloric acid containing 0.1% phenol.

Next, 100 ml of 6N hydrochloric acid (constant boiling HC1, supplied by BDH, Dorset, UK) containing 0·1% phenol are added, together with anti-bumping granules. Samples are refluxed for 20 h at 110 °C on a multi-place heating mantle. After cooling, the contents of each flask are quantitatively transferred to a 200-ml volumetric flask and made up to the mark. After thorough mixing, the solutions are filtered through GF/A grade glass fibre filter paper.

*Sep-pak clean-up procedure*

Waters Associates recommend a clean-up procedure for hydrolysates in order to remove high molecular weight proteins and lipids from samples prior to amino-acid analysis. One method suggested involves passing the sample through a Sep-pak C18 cartridge after suitable preparation of the latter. Lipids and high molecular weight proteins should be retained on the Sep-pak cartridge, while the amino-acid fraction elutes.

For Sep-pak clean-up, the following solutions are required:

Solution 1 — 0·1% (V/V) trifluoroacetic acid (TFA) in distilled water

Solution 2 — 0·1% (V/V) TFA in water: methanol, 80:20 (V/V)

Solution 3 — 0·1% (V/V) TFA in water: methanol, 70:30 (V/V).

For each sample, a new Sep-pak C18 cartridge is activated with two 10 ml volumes of methanol, washed with two 10 ml volumes of solution 1 and then with 10 ml of solution 2.

A 1 ml sample is then mixed with 2 ml of solution 3 and passed through the activated cartridge. The first 1 ml of eluent is discarded and the next 2 ml are collected. This fraction contains the amino-acids, ready for derivatization.

*Drying and derivatization*

Aliquots of hydrolysate (typically 5–20 µl), together with appropriate standards, are placed in disposable glass sample tubes (6 × 50 mm, Pyrex, from Waters Associates or 7 × 50 mm, soda glass from Fisons), which in turn are loaded into a reaction vial. Up to 12 of the smaller sample tubes can be processed together in one vial. These vials feature a vacuum-tight top with a drilled-through Teflon valve. Opening and closing the vials to the vacuum is accomplished by pushing the 'stop-go' slider button at the top.

Hydrochloric acid is removed from the samples by drying under vacuum at room temperature, achieved by connecting the vial to the work station manifold and opening the vacuum control valve. The samples are properly evaporated when the reading on the vacuum gauge reaches about 65 millitorr. In practice, values lower than this are easily achieved with the pump used in our system, namely an E2M5 model two-stage rotary vacuum pump (Edwards High Vacuum, Crawley, UK).

Samples are then redried from 10 μl redrying reagent (table 1), again using the work station. Derivatization is initiated by addition of 20 μl of freshly prepared reagent (table 1), which is mixed using a vortex mixer and allowed to stand at room temperature for 20 min. The entire reagent is then removed under vacuum using the work station. It is essential to dry the samples thoroughly at this stage, to remove excess reagent and by-products, which give interfering peaks in the chromatogram. In this dried state, derivatized samples may be stored at freezer temperatures for several weeks, if required, before analysis.

PTC derivatives are made ready for analysis by being dissolved in sample diluent (see table 1). It is recommended that, once dissolved, they should be analysed within 10 h if kept at room temperature, or within 60–70 h if kept refrigerated. Such solutions have, however, been analysed after 16 h at room temperature with no apparent losses.

*Table 1. Composition of reagents used with the Pico-Tag system.*

| Reagent | Composition (by volume) |
|---|---|
| Redrying | Ethanol 95 : Water : triethylamine (2:2:1) |
| Derivatization | Ethanol 95 : water : triethylamine : phenylisothiocyanate (7:1:1:1) |
| Sample diluent | 5 mM sodium phosphate ($Na_2HPO_4$) buffer, pH 7.4 : acetonitrile (95:5) |

Protein hydrolysate calibration mixes of amino-acids are available commercially, for example from Pierce UK Ltd. A useful concentration of amino-acids is 2·5 μmol/ml, with cystine present at 1·25 μmol/ml. An aliquot of this calibration mix is mixed with a norleucine solution in 0.1 N HC1 (prepared in the laboratory) to give a standard mixture containing 2 μmol/ml of each amino-acid, including the internal standard norleucine. Cystine is present at 1 μmol/ml. For each batch of samples processed on the work station, one or two standards (5 μl) are also included. A standard chromatogram (see figures 2[*a*], 3[*a*], 4[*a*], 5[*a*], and 6) represents 500 pmol of each amino-acid (250 pmol of cystine) actually injected.

*Separation*

Separation of the PTC derivatives takes place by high-performance liquid chromatography. The column is a reversed-phase Nova-Pak C18, 3·9 × 150 mm, specially selected and tested for suitability in separating PTC-amino-acids. It is maintained at 38 °C (± 1 °C) in the column heater.

The mobile phase consists of two eluents labelled A and B. A comprises 940 ml of 0·14 M sodium acetate, pH 6·40, containing 0·05% triethylamine, mixed with 60 ml acetonitrile. B is 60% acetonitrile and 40% water by volume. A standard gradient elution programme, recommended by Waters with eluent B increasing, was employed for the work reported here.

An additional step of 100% eluent B is used to wash the column prior to returning to initial conditions.

Both eluents may either be purchased from Waters Associates or prepared in the laboratory. The manufacturer recommends the use of water with a resistivity of at least 18 megohm/cm.

The quality of the water used is quite an important factor. According to the manufacturers, histidine peak shape can be affected by the presence of amines in the water. In the absence of water of known resistivity, distilled would normally be satisfactory. De-ionized water is not acceptable because it contains organic compounds which alter column selectivity.

Detection of the PTC derivatives is by ultraviolet absorption measurements using a fixed wavelength (254 nm) detector. The λ max of the derivatives is 269 nm, but detection at 254 is quite satisfactory. All of the common amino-acids are eluted in 12 min; lysine elutes last. An 8 min equilibration delay time is used at the end of each run, giving a total analysis time of 20 min for each sample. In practice, it has been found that for certain samples analysed on the Pico-Tag, successive chromatograms contain shifts and definite humps in the base-line. This contamination may be due to incomplete cleaning of the column between samples and can be avoided by including a 5 min wash with 100% B at the end of the run. This increases the equilibration delay time from 8 to 13 min, thus giving a total analysis time of 25 min.

*Free amino-acid analysis*

The analysis of free amino-acids has also been demonstrated using the Pico-Tag analyser with encouraging results. Free amino-acids are normally extracted from foods using hot aqueous acid (for example $5 \times 10^{-3}$ N hydrochloric at 80 °C). After deproteination of the extract with sulphosalicylic acid, aliquots are derivatized on the Pico-Tag work station as previously described.

During preparation of samples for free amino-acid analysis, the addition of sample diluent to the dried, derivatized samples results in the formation of a cloudy white solution. When this occurs, the tubes are centrifuged at 10 000 rev/min for 10 min and the resulting clear supernatants are taken for analysis.

*Food analysed in this investigation*

The following food materials were analysed for total amino-acids on the Pico-Tag:

(1) Bipro (whey protein isolate), No. 3131 from Bioisolates, Swansea, UK. Samples of Bipro were analysed on an LKB 4400 Amino Acid Analyser (an IEC system which has post-column derivatization with ninhydrin) using sodium salt buffers, as well as on the Pico-Tag analyser with and without the Sep-pak clean-up. Four replicate samples were hydrolysed.

(2) Soya molasses—an example of a food material containing vegetable protein. This sample was analysed by both the IEC and Pico-Tag methods.

(3) Infant liquid and milk powder formulations—examples of foods containing dairy proteins.

*Table 2. Amino-acid composition of Bipro from the Pico-Tag analyser (with and without Sep-pak clean-up) and an IEC system.*

Results are expressed in milligrams per gram of protein (protein content N × 6·38 = 93·8%)

| Amino-acid | Composition from Pico-Tag +mean | ++S.D. | Composition from IEC system +mean | ++S.D. | Approx. value from literature | Apparent composition from Pico-Tag after Sep-pak clean-up +mean | ++S.D. |
|---|---|---|---|---|---|---|---|
| ASP | 124 | 3 | 117 | 4 | 132 | 167 | 7 |
| GLU | 196 | 3 | 191 | 5 | 177 | 258 | 8 |
| SER | 42·5 | 0·6 | 46·3 | 2·1 | 42·0 | 68·9 | 3·6 |
| GLY | 18·9 | 0·9 | 21·6 | 2·4 | 18·5 | 27·6 | 1·5 |
| HIS | 19·4 | 2·0 | 22·5 | 2·6 | 19·3 | 23·8 | 2·1 |
| ARG | 33·6 | 1·5 | 27·2 | 4·5 | 24·0 | 48·8 | 2·0 |
| THR | 59·9 | 1·8 | 54·4 | 2·2 | 51·3 | 86·8 | 4·0 |
| ALA | 64·4 | 2·1 | 62·2 | 4·0 | 57·8 | 90·7 | 3·7 |
| PRO | 52·5 | 1·8 | 53·4 | 4·0 | 42·0 | 69·1 | 3·2 |
| TYR | 44·4 | 0·9 | 44·2 | 3·0 | 41·3 | 49·1 | 3·1 |
| VAL | 59·1 | 1·4 | 56·7 | 2·1 | 57·5 | 78·9 | 2·9 |
| MET | 22·2 | 0·5 | 29·2 | 1·2 | 26·4 | 30·1 | 1·0 |
| CYS | 17·6 | 1·0 | 27·3 | 2·0 | 41·5 | 27·0 | 4·0 |
| ILE | 59·0 | 2·0 | 53·1 | 0·2 | 68·8 | 64·9 | 1·0 |
| LEU | 151 | 2 | 127 | 9 | 145 | 160 | 3 |
| PHE | 40·0 | 2·0 | 40·8 | 5·0 | 37·5 | 35·2 | 1·7 |
| LYS | 112 | 4 | 110 | 11 | 117 | 152 | 9 |
| Total | 1117 | | 1084 | | 1099 | 1438 | |

\+ Mean of four determinations
\++ Standard deviation, $\sigma_{n-1}$

(4) Drinde animal protein (a dehydrated, sterilized meat protein powder produced from pork rind) coded standard 1020/4 from Protein Foods, Scandinavia A/S. This material contains quite a high proportion of hydroxyproline. The results obtained from the Pico-Tag were compared with those from the published specification for the material.

### Results

### *Bipro*

Results for the analysis of Bipro on the Pico-Tag, with and without sample clean-up and on an IEC system are given in table 2. Also included in the table are the expected values for the amino-acid composition from the literature [4]. These values represent weighted averages on the basis that WPI consists essentially of β-lactoglobulin and α-lactalbumin in the ratio of 3 to 1.

Bipro results were in line with the expected composition of whey protein isolate, provided that no Sep-pak clean-up was employed. The results are patently wrong if the clean-up procedure is used, the total amino-acid content appearing to be considerably higher than is theoretically possible. The Pico-Tag chromatography was good enough for automated integration/calculation in practically all cases. There was also good agreement between the results obtained for a Bipro sample analysed on the IEC and Pico-Tag analysers.

Figure 2 illustrates typical chromatograms for a standard amino-acid mixture, together with a Bipro sample.

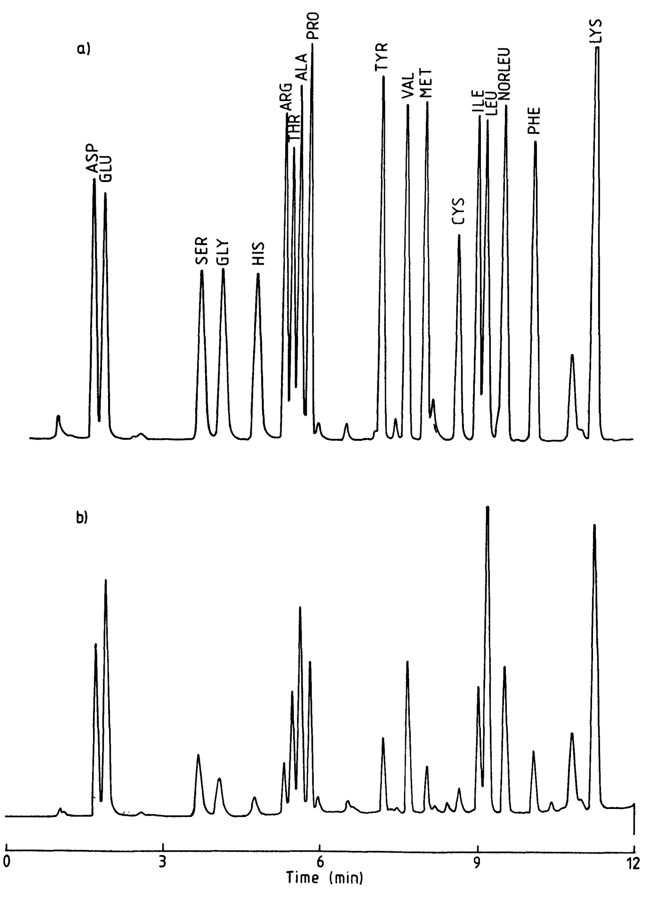

*Figure 2. Pico-Tag profiles of (a) standard amino-acid mixture and (b) whey protein isolate.*

*Table 3. Amino-acid composition of soya molasses from the Pico-Tag analyser (with and without Sep-pak clean-up) and an IEC system. Concentrations are in milligrams per gram of product. Results are the means of duplicate injections*

| Amino-acid | Composition from IEC system | Composition from Pico-Tag | Apparent composition from Pico-Tag after sample clean-up |
|---|---|---|---|
| ASP | 6·0 | 5·5 | 7·3 |
| GLU | 8·0 | 8·7 | 10·7 |
| SER | 1·7 | 1·8 | 2·3 |
| GLY | 1·9 | 1·9 | 2·4 |
| HIS | 1·4 | 1·9 | 2·7 |
| ARG | 2·9 | 3·5 | 4·5 |
| THR | 1·8 | 2·0 | 2·6 |
| ALA | 2·0 | 2·1 | 2·6 |
| PRO | 2·0 | 2·1 | 2·6 |
| TYR | 2·2 | 2·2 | 2·9 |
| VAL | 1·4 | 1·4 | 1·6 |
| MET | 0·9 | 1·8 | 2·2 |
| CYS | 1·3 | 0·4 | 0·5 |
| ILE | 1·3 | 1·4 | 1·3 |
| LEU | 1·9 | 1·9 | 1·9 |
| PHE | 2·2 | 2·3 | 1·7 |
| LYS | + | ++ | 1·4 |

+ impossible to quantify as co-eluted with other ninhydrin positive compound.

++ cannot be quantified due to shoulder on peak.

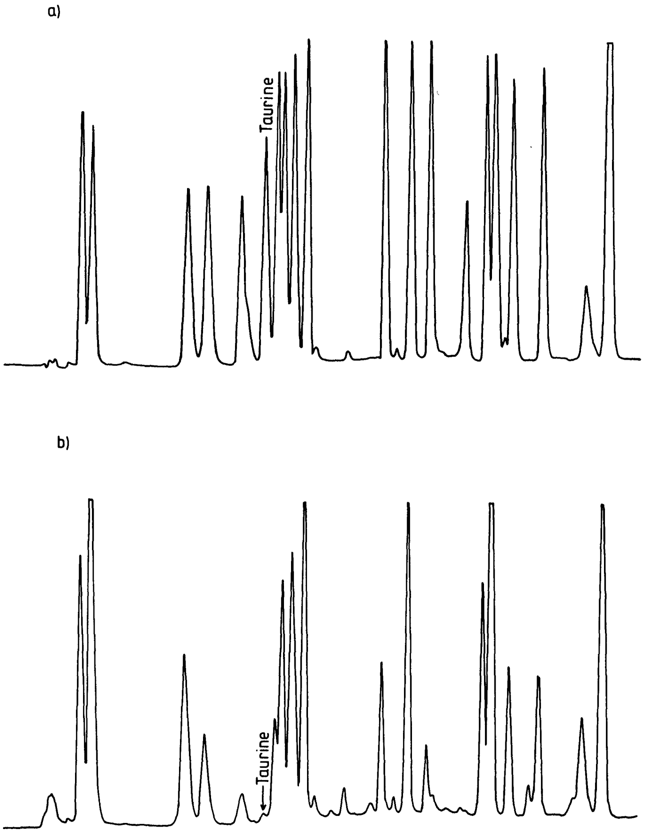

*Figure 3. Pico-Tag profiles of (a) standard amino-acid mixture including taurine and (b) infant liquid milk formulation.*

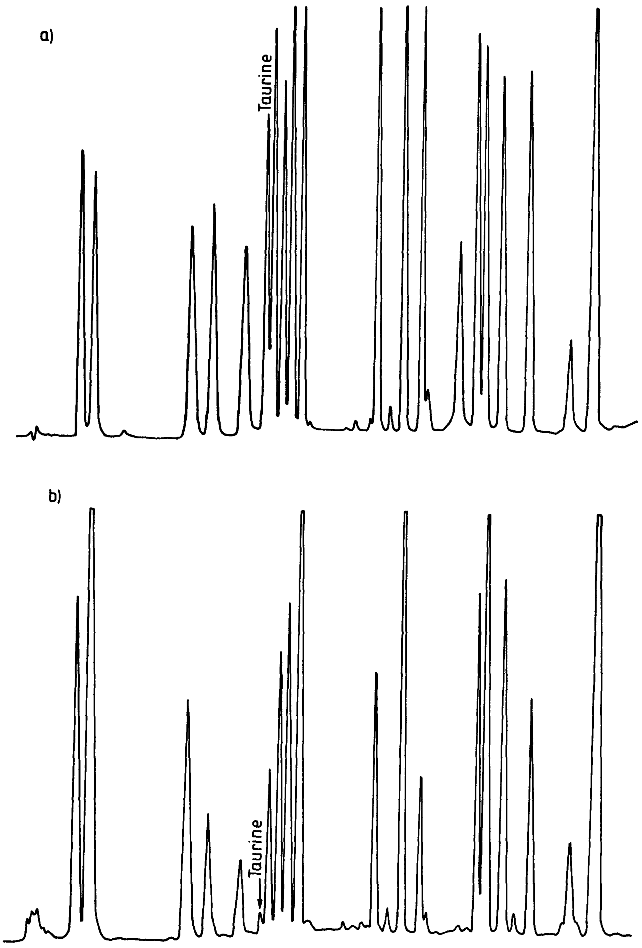

*Figure 4. Pico-Tag profiles of (a) standard amino-acid mixture including taurine and (b) milk powder formulation.*

*Table 4. Amino-acid composition of Drinde. Concentrations are expressed in milligrams of amino-acid per gram of product.*

| *Amino-acid* | **Composition from Pico-Tag* | *Value from data sheet* |
|---|---|---|
| ASP | 51·2 | 54·5 |
| GLU | 86·3 | 84·4 |
| HO-PRO | 79·2 | 69·9 |
| SER | 28·0 | 24·5 |
| GLY | 167·4 | 167·5 |
| HIS | 7·9 | 9·6 |
| ARG | 62·5 | 63·0 |
| THR | 15·8 | 16·3 |
| ALA | 68·3 | 65·6 |
| PRO | 103·3 | 103·3 |
| TYR | 9·7 | 10·5 |
| VAL | 23·4 | 23·8 |
| MET | 9·8 | 5·3 |
| CYS | 3·0 | 3·0 |
| ILE | 13·9 | 12·5 |
| LEU | 31·6 | 29·4 |
| PHE | 23·9 | 20·0 |
| LYS | 29·8 | 33·6 |
| Totals: | 815 | 797 |

* results are the means of triplicate injections.

*Soya molasses*

Results for the analysis of soya molasses are given in table 3. A comparison is made between the results obtained from the IEC system and from the Pico-Tag, with and without sample clean-up.

Again LKB and Pico-Tag results agreed well, but after Sep-pak clean-up results were higher in most cases. In view of the effect of sample clean-up on the previous analyses, the results are suspicious.

Retention times varied slightly from standard, necessitating manual calculation of results. Sample clean-up alleviated this problem but, as explained, the results are suspect.

*Infant milk formulations*

The chromatography was good for both liquid milks and milk powder formulations. The majority of peaks were recognized correctly. The analyses demonstrated that taurine can be successfully determined; the peak elutes between histidine and arginine. Representative chromatograms of a standard and a baby milk (approximately 2% protein) are given in figure 3, and for a milk powder formulation (approximately 13% protein) in figure 4.

*Drinde animal protein*

Results for the analysis of Drinde on the Pico-Tag are given in table 4, together with the value from the specification sheet.

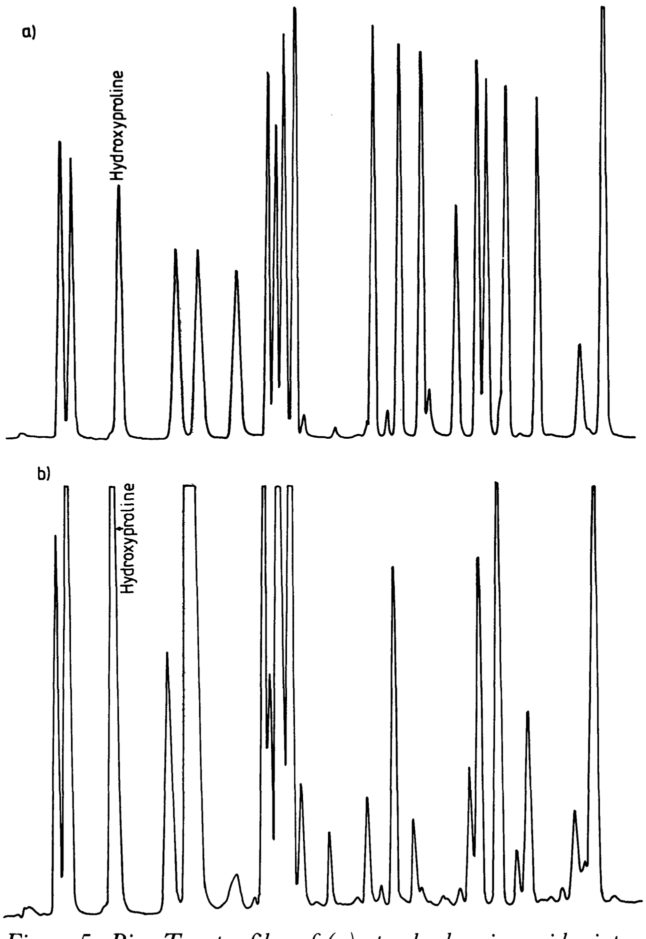

*Figure 5. Pico-Tag profiles of (a) standard amino-acid mixture including hydroxyproline and (b) Drinde animal protein.*

The internal standard was identified correctly in most runs. The results agreed well with the manufacturer's specifications sheet. The sample contained quite a high proportion of hydroxyproline (7%), which could easily be measured on the Pico-Tag using the normal standard gradient elution program. The peak elutes almost midway between glutamic acid and serine and is, consequently, very well resolved. This is in contrast to the IEC system used in this work, where a modified program (i.e. four sodium salt buffers instead of three, and a longer equilibration time) is required to achieve separation of hydroxyproline.

Figure 5 illustrates Pico-Tag profiles for a standard amino-acid mixture containing hydroxyproline and a sample of Drinde.

### Discussion

*Comparison of analysis times and chromatography between Pico-Tag and conventional ion-exchange separation*

Depending on the diameter of the glass sample tubes used, batches of up to 12 can be prepared on the Pico-Tag work station at one time. The samples, together with the appropriate standards, are dried, re-dried and derivatized as a batch; the whole process takes about 3–3½ h. The operator would actually be involved for approximately 1 h of the total processing time.

Preparation of samples for analysis by ion-exchange chromatography involves rotary evaporation of single samples to dryness, addition of loading buffer to the flasks and thorough dispersion of the residues. Filtering of the samples through millipore membranes is then essential. For the preparation of 12 samples about 4½ h are required. This represents total involvement of the operator.

As mentioned earlier, analysis times with the Pico-Tag are very short; typically, only a 12 min run time is required with an 8 min equilibration, which can be increased to 13 min if required. However, the IEC system employed in this study required run times of 75 min (i.e. more than six times longer than the Pico-Tag analysis time), followed by a regeneration and equilibration period of about 13 min. The chromatography obtained with the Pico-Tag is excellent, with clear advantages over that produced from IEC. Most notable features, in addition to faster analysis times, are excellent base-line stability, very good resolution and sharper peaks with very consistent retention times, which allow automatic peak integration and calculation. This feature of the Pico-Tag is in contrast to the IEC system, where run-to-run variations in retention time can make it difficult to achieve successful fully automatic operation and integration. Multiple injections of samples, which are generally very reproducible, are routinely made on the Pico-Tag system.

*General discussion*

Good agreement was obtained for the amino- acid analysis results of Bipro and soya molasses between Pico-Tag and ion-exchange methods.

Taurine was analysed successfully in milk formations (powder and liquid) and was better resolved than with the IEC method, when it often co-eluted with a peak which had a strong absorbance at 440 nm.

Hydroxyproline in a meat product was readily determined using the standard gradient program. Greater accuracy would be expected for proline and hydroxyproline than with conventional ninhydrin detection, which gives a small response, and hence a potential loss in accuracy, for these amino-acids.

Samples can quite readily be analysed for free amino-acids, a service which food manufacturers frequently require. Generally, the chromatography is excellent for free amino-acid analyses. A peak resolution similar to and generally better than that obtained by IEC has been observed. A feature of Pico-Tag chromatograms for free amino-acid analyses is the presence of a peak (eluted at about 1.1–1.2 min) for residual sulphosalicylic acid. This peak, although quite large, does not generally interfere with the elution of the first two amino-acid peaks, viz. aspartic and glutamic acids. More work would, however, be required before the use of sulphosalicylic acid could be recommended as excess reagent may interfere with the derivatization yield.

Samples can be analysed routinely on the Pico-Tag using multiple injections. In most cases, the replicates are in excellent agreement, which, in turn, provides greater confidence in results.

In the authors' laboratory, the Waters Pico-Tag is used exclusively for all amino-acid analysis work and has proved to be extremely reliable.

While the Pico-Tag technique, as it stands, is successful, we must express some concern over the use of the Sep-pak system for sample clean-up. Our experiments have demonstrated that the wrong results can be obtained after this procedure. This would be the subject of a further publication; at present there is no clean-up procedure being used.

In addition to the detailed results given above, the Pico-Tag has been used for the amino-acid analysis of a wide variety of foods, including canned 'convenience' foods, for example baked beans and sausages; fruit juices; bone, meat and carcase meals; seeds and canned and dried pet foods. As well as those amino-acids already mentioned, cysteic acid and methionine sulphone have been successfully determined. These compounds are formed during performic acid oxidation treatment and are stable to acid hydrolysis. As cystine and methionine are susceptible to degradation on normal acid hydrolysis of foods, their accurate determination requires pre-treatment with performic acid (to convert them to their stable oxidized forms) followed by hydrolysis.

A standard chromatogram is given in figure 6, where the positions of cysteic acid and methionine sulphone can be seen in relation to the standard amino-acids.

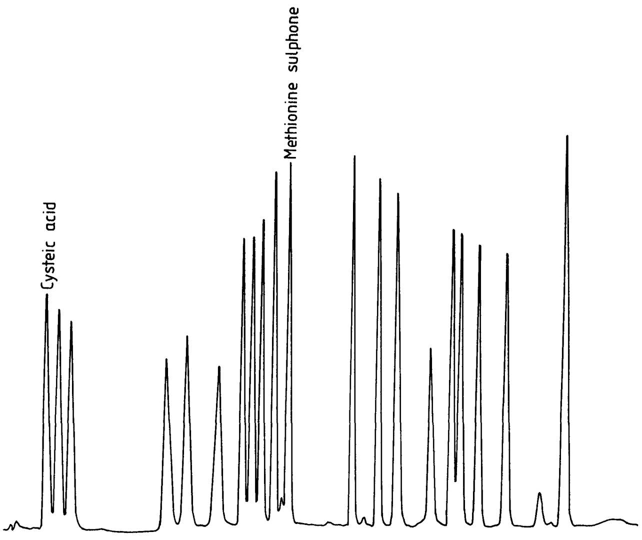

*Figure 6. Standard calibration amino-acid chromatogram with the additon of cysteic acid and methionine sulphone.*

The determination of cysteic acid and methionine sulphone requires a slightly modified programme. The pH of eluent A is changed to 6·1 (normal programme requires 6·4), but the actual gradient during analysis remains similar. As eluent A at pH 6·1 gives excellent resolution of all common amino-acids, it has now been adopted for all routine analyses in the authors' laboratory.

### Costs

The price of a complete Waters Pico-tag system (at July 1986) is £27 202. This includes items 1–8 in the description of the system (above) a 'chemistry package' of eluants, reagents etc; start-up by Waters personnel and one free place on a Waters training course. Twelve months' warranty (parts, labour and travel) is also included, except for some specified unwarrantable parts.

Enquiries of three manufacturers of comparable ion exchange systems for amino-acid analysis revealed prices ranging from £25–£40 000; the Pico-tag system is quite reasonably placed within this range.

### Conclusion

The Pico-Tag amino-acid analysis system has been used to determine the amino-acid composition of a variety of foods with both speed and accuracy. Results agree well with the literature and with those obtained by conventional ion-exchange chromatography.

The advantages of the Pico-Tag claimed by its manufacturer appear to be fully justified.

The Pico-Tag methodology would then appear to be a real alternative to ion-exchange separation with postcolumn derivatization for amino-acid analysis. It has proved to be well-suited to the analysis of foods.

### Acknowledgement

This work forms part of Project M48, 'Carbohydrate—protein interactions during the thermal processing of foods', funded by the Ministry of Agriculture, Fisheries and Foods.

### References

1. HEINRIKSON, R. L. and MEREDITH, S. C., *Analytical Biochemistry*, **136** (1984), 65.

2. BIDLINGMEYER, B. A., COHEN, S. A. and TARVIN, T. L., *Journal of Chromatography*, **336** (1984), 93.

3. MOORE, S., SPACKMAN, D. H. and STEIN, W. H., *Analytical Chemistry*, **30** (1958), 1185.

4. GORDON, W. G. and WHITTIER, E. O., *Fundamentals of Dairy Chemistry*, Ed. Webb, B. H. and Johnson, A. H. (The AVI Publishing Co, 1965), 60.

### ENDOWMENT FUND CREATED TO HONOR THE LATE TOMAS HIRSCHFELD

In memory of Tomas Hirschfeld, and his contributions to applied chemistry, the Center for Process Analytical Chemistry at the University of Washington has created a special endowment fund. In addition to Tomas's position as Senior Staff Scientist at Lawrence Livermore National Laboratory, he was a CPAC researcher and an Affiliate Professor in the Department of Chemistry at the University of Washington.

Contributions to the fund are being handled by the Gift Processing Office at the University of Washington, AJ-55, Seattle, WA 98195. According to Deborah Illman, Assistant Director of CPAC, one of the things the endowment will support is a special graduate student fellowship at CPAC.

Hirschfeld will be remembered for his contributions to the fields of spectroscopy, fibre optics and their use in process analytical chemistry. Evidence of the importance of these contributions comes from the numerous awards he received in his 25-year career including: the Meggar's Award (Society for Applied Spectroscopy, 1978); the Louis A. Strait Award (Society for Applied Spectroscopy, 1984); the Pittsburgh Spectroscopy Award (1986); IR-100 Awards (1975, 1977, 1981, 1983, 1985).

### NOTES FOR AUTHORS

*Journal of Automatic Chemistry incorporating Journal of Clinical Laboratory Automation* covers all aspects of automation and mechanization in analytical, clinical and industrial environments. The *Journal* publishes original research papers; short communications on innovations, techniques and instrumentation, or current research in progress; reports on recent commercial developments; and meeting reports, book reviews and information on forthcoming events. All research papers are refereed.

### Manuscripts

Two copies of articles should be submitted. All articles should be typed in double spacing with ample margins, on one side of the paper only. The following items should be sent: (1) a title-page including a brief and informative title, avoiding the word 'new' and its synonyms; a full list of authors with their affiliations and full addresses; (2) an abstract of about 250 words; (3) the main text; (4) appendices (if any); (5) references; (6) tables, each table on a separate sheet and accompanied by a caption; (7) illustrations (diagrams, drawings and photographs) numbered in a single sequence from 1 upwards and with the author's name on the back of every illustration; captions to illustrations should be typed on a separate sheet. Papers are accepted for publication on condition that they have been submitted only to this *Journal.*

### References

References should be indicated in the text by numbers following the author's name, i.e. Skeggs [6]. In the reference section they should be arranged thus:

to a journal

MANKS, D. P., *Journal of Automatic Chemistry*, **3** (1981), 119.

to a book

MALMSTADT, H. V., in *Topics in Automatic Chemistry*, Ed. Stockwell, P. B. and Foreman, J. K. (Horwood, Chichester, 1978), p. 68.

### Illustrations

Original copies of diagrams and drawings should be supplied, and should be drawn to be suitable for reduction to the page or column width of the *Journal*, i.e. to 85 mm or 179 mm, with special attention to lettering size. Photographs may be sent as glossy prints or as negatives.

### Proofs and offprints

The principal or corresponding author will be sent proofs for checking and will receive 50 offprints free of charge. Additional offprints may be ordered on a form which accompanies the proofs.

*Manuscripts should be sent to either Dr P. B. Stockwell or Ms M. R. Stewart, see inside front cover.*

